# Achieving a steady pulse with pulse field ablation

**DOI:** 10.1007/s10840-024-01902-y

**Published:** 2024-08-17

**Authors:** Rachel M. Kaplan, Matthew Long, Sergio L. Pinski

**Affiliations:** Division of Cardiac Electrophysiology, Medical University of South Carolina, Charleston, SC USA

A 76-year-old man with a history of hypertension and prior left atrial appendage occlusion device presented with recurrent atypical atrial flutter with rapid ventricular rates. He had undergone two prior ablations. His first ablation was 6 years earlier and involved a radiofrequency wide area circumferential ablation for atrial fibrillation. His second ablation was 1 year prior to his current presentation and involved redo isolation of the right pulmonary veins in addition to radiofrequency ablation of peri-mitral flutter. The flutter was terminated with extensive endocardial ablation on the inferior/lateral mitral isthmus, but block was not achieved at that time.

**Fig. 1:**
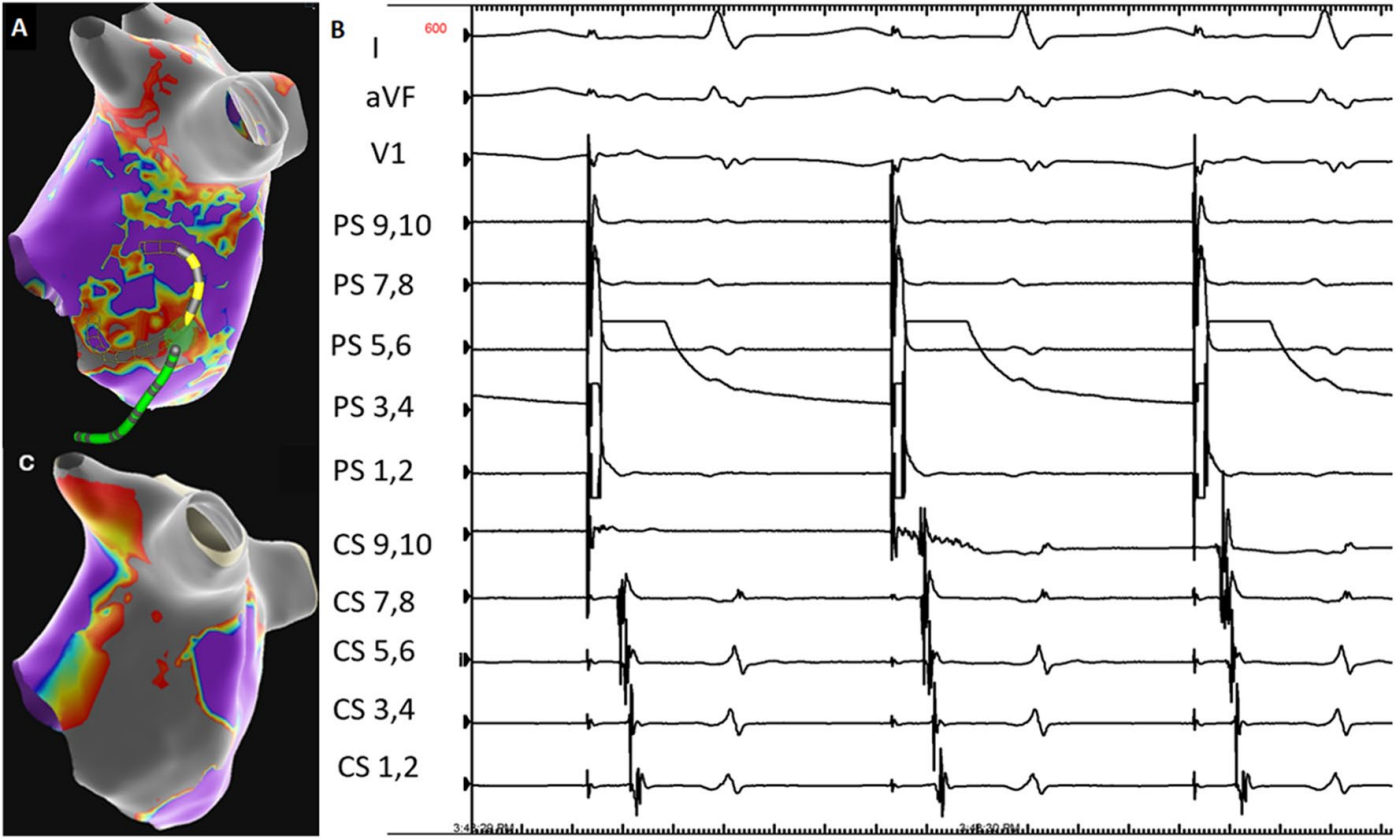
**A** High-density voltage map prior to repeat ablation with example positioning of PulseSelect PFA catheter. **B** Pacing from anterior to the mitral line via the PulseSelect PFA catheter after ablation. **C** High-density voltage map performed post-ablation

The patient presented to the electrophysiology lab in atrial flutter with a cycle length of 225 ms. Activation mapping demonstrated a roof-dependent flutter which transitioned to cavotricuspid isthmus flutter after pulsed field ablation of a roof line. The flutter terminated with radiofrequency ablation of the cavotricuspid isthmus. The mitral line was checked once sinus rhythm restored. Pacing anterior to the prior mitral line demonstrated that it was not blocked. Voltage map revealed heterogenous voltage at the location of prior endocardial radiofrequency ablation (Fig. 1A).

Considering the prior unsuccessful endocardial radiofrequency ablation at the mitral isthmus, pulse field ablation was used in hopes of creating a more transmural lesion to achieve block at the mitral isthmus. The circular array pulsed field ablation (PFA) catheter (PulseSelect, Medtronic, USA) was then positioned at the lateral mitral isthmus after advancing the guiding wire across the mitral valve. Eight energy deliveries were performed with slight rotation of the catheter to maintain it along the line (Fig. 1A). The catheter was then moved anterior again and pacing now demonstrated the presence of block across the line (Fig. 1B).

High-density mapping post-ablation demonstrated a new wide area of electric silence along the mitral isthmus line (Fig. 1C).

Mitral isthmus block is notoriously difficult to achieve with endocardial radiofrequency ablation on the lateral mitral isthmus. Epicardial ablation via the coronary sinus is often required and vein of Marshall ethanol infusion may also be needed. Use of a pentaspline pulsed field ablation (PFA) catheter for mitral isthmus ablation has been described by Kueffer et al. [1]; however, this is the first reported case of using the circular array PFA catheter after prior failed radiofrequency ablation. PFA may be able to achieve a more transmural lesion compared to radiofrequency in difficult areas like the lateral mitral isthmus [2]. Studies of PFA over areas of prior radiofrequency ablation in the left atrium have shown that PFA remains effective despite prior ablation [3]. Durability of the lesion with endocardial PFA remains to be seen, though this patient has had no clinical recurrence of atrial flutter to date.

Vasospasm of the circumflex coronary artery has been reported with PFA from the pentaspline catheter on the mitral isthmus [4]. In this case, a 1-mg bolus of nitroglycerin was administered immediately prior to PFA on the mitral isthmus. No clinical coronary spasm was observed.

This is the first reported case of using the circular array PFA catheter for mitral isthmus ablation after failed endocardial radiofrequency ablation. PFA may represent an efficient option for achieving mitral isthmus block using exclusively endocardial ablation.
